# A hybrid model‐ and deep learning‐based framework for functional lung image synthesis from multi‐inflation CT and hyperpolarized gas MRI

**DOI:** 10.1002/mp.16369

**Published:** 2023-04-01

**Authors:** Joshua R. Astley, Alberto M Biancardi, Helen Marshall, Paul J. C. Hughes, Guilhem J. Collier, Matthew Q. Hatton, Jim M. Wild, Bilal A. Tahir

**Affiliations:** ^1^ Department of Oncology and Metabolism The University of Sheffield Sheffield UK; ^2^ POLARIS, Department of Infection, Immunity & Cardiovascular Disease The University of Sheffield Sheffield UK; ^3^ Insigneo Institute for In Silico Medicine The University of Sheffield Sheffield UK

**Keywords:** Functional lung imaging, CT ventilation, Hyperpolarized gas MRI, Deep learning, Image synthesis

## Abstract

**Background:**

Hyperpolarized gas MRI is a functional lung imaging modality capable of visualizing regional lung ventilation with exceptional detail within a single breath. However, this modality requires specialized equipment and exogenous contrast, which limits widespread clinical adoption. CT ventilation imaging employs various metrics to model regional ventilation from non‐contrast CT scans acquired at multiple inflation levels and has demonstrated moderate spatial correlation with hyperpolarized gas MRI. Recently, deep learning (DL)‐based methods, utilizing convolutional neural networks (CNNs), have been leveraged for image synthesis applications. Hybrid approaches integrating computational modeling and data‐driven methods have been utilized in cases where datasets are limited with the added benefit of maintaining physiological plausibility.

**Purpose:**

To develop and evaluate a multi‐channel DL‐based method that combines modeling and data‐driven approaches to synthesize hyperpolarized gas MRI lung ventilation scans from multi‐inflation, non‐contrast CT and quantitatively compare these synthetic ventilation scans to conventional CT ventilation modeling.

**Methods:**

In this study, we propose a hybrid DL configuration that integrates model‐ and data‐driven methods to synthesize hyperpolarized gas MRI lung ventilation scans from a combination of non‐contrast, multi‐inflation CT and CT ventilation modeling. We used a diverse dataset comprising paired inspiratory and expiratory CT and helium‐3 hyperpolarized gas MRI for 47 participants with a range of pulmonary pathologies. We performed six‐fold cross‐validation on the dataset and evaluated the spatial correlation between the synthetic ventilation and real hyperpolarized gas MRI scans; the proposed hybrid framework was compared to conventional CT ventilation modeling and other non‐hybrid DL configurations. Synthetic ventilation scans were evaluated using voxel‐wise evaluation metrics such as Spearman's correlation and mean square error (MSE), in addition to clinical biomarkers of lung function such as the ventilated lung percentage (VLP). Furthermore, regional localization of ventilated and defect lung regions was assessed via the Dice similarity coefficient (DSC).

**Results:**

We showed that the proposed hybrid framework is capable of accurately replicating ventilation defects seen in the real hyperpolarized gas MRI scans, achieving a voxel‐wise Spearman's correlation of 0.57 ± 0.17 and an MSE of 0.017 ± 0.01. The hybrid framework significantly outperformed CT ventilation modeling alone and all other DL configurations using Spearman's correlation. The proposed framework was capable of generating clinically relevant metrics such as the VLP without manual intervention, resulting in a Bland‐Altman bias of 3.04%, significantly outperforming CT ventilation modeling. Relative to CT ventilation modeling, the hybrid framework yielded significantly more accurate delineations of ventilated and defect lung regions, achieving a DSC of 0.95 and 0.48 for ventilated and defect regions, respectively.

**Conclusion:**

The ability to generate realistic synthetic ventilation scans from CT has implications for several clinical applications, including functional lung avoidance radiotherapy and treatment response mapping. CT is an integral part of almost every clinical lung imaging workflow and hence is readily available for most patients; therefore, synthetic ventilation from non‐contrast CT can provide patients with wider access to ventilation imaging worldwide.

## 1 INTRODUCTION

Lung diseases represent significant global health challenges.^1^, ^2^ Imaging of the lungs constitutes a key component of clinical care, providing both anatomical and functional information for a wide range of lung pathologies. Functional lung imaging modalities such as single‐photon emission computed tomography (SPECT), positron emission tomography (PET) and hyperpolarized gas magnetic resonance imaging (MRI) have shown efficacy in several applications such as early diagnosis, functional lung avoidance radiotherapy and treatment response evaluation.^3^, ^4^, ^5^ Hyperpolarized gas MRI is a functional lung imaging modality capable of visualizing regional lung ventilation with exceptional detail within a single breath.^6^ Quantitative biomarkers derived from this modality, including the ventilated lung percentage (VLP), provide further insights into regional ventilation.^7^ However, this modality requires specialized equipment, including a laser polarizer, and inhaled contrast agents such as helium‐3 (^3^He) or xenon‐129 (^129^Xe) noble gases, which currently limits widespread clinical adoption.^8^

Computed tomography (CT) is the most widely used anatomical imaging modality and is an integral part of clinical care for most patients with lung pathologies. CT ventilation imaging (CTVI) aims to model regional ventilation from non‐contrast CT scans acquired at multiple inflation levels, either during tidal breathing or breath‐hold.^9^, ^10^ CTVI assumes that changes in regional lung volume and/or lung density between inflation levels is representative of lung ventilation.^11^ Several metrics have been proposed to generate synthetic ventilation maps from multi‐inflation CT, such as those that map changes in Hounsfield units (CT^HU^) or the determinant of the Jacobian (CT^JAC^).^9^, ^10^ The CT^HU^ metric is based on differences in HU intensities between inflation levels whereas the CT^JAC^ metric is a measure of volume expansion computed directly on the deformation vector field between inflations. Previous validation of CTVI methods included assessing Spearman's correlation with well‐established lung function measures, such as spirometry, resulting in moderate correlations, ranging from 0.38 to 0.73 for both the CT^HU^ and CT^JAC^ methods.^12^, ^13^ CTVI models have also been validated against nuclear imaging modalities, exhibiting moderate correlation with SPECT and PET imaging^14^, ^15^; however, these studies report highly variable results and often use small numbers of patients.^16^ Furthermore, nuclear medicine imaging has a relatively poor spatial and temporal resolution and a susceptibility to aerosol deposition artifacts, particularly within defect regions.^17^, ^18^ In addition, the requirement of radioactive contrast agents makes nuclear medicine imaging unattainable for some patient groups, for example, pediatrics. By using hyperpolarized gas MRI for validation, Tahir et al.^19^ showed moderate Spearman's correlations of several CTVI metrics.

Recently, deep learning (DL)‐based methods utilizing convolutional neural networks (CNNs) have become widespread in numerous lung imaging applications, including image synthesis.^20^ Zhong et al.^21^ used a CNN to synthesize CT‐based ventilation surrogates from 4DCT, reporting a mean square error (MSE) of 7.6%. However, a limitation of this approach is that CT ventilation images, used as the ground truth ventilation, are in themselves the subject of intense validation efforts.^22^ Ren et al.^23^ have shown the capability of deriving synthetic perfusion maps from CT using SPECT perfusion as ground truth; a 3D UNet CNN was used, achieving an average Spearman's correlation of 0.81 using three‐fold cross‐validation. A Dice similarity coefficient (DSC) value of 0.81 was achieved for both high‐functional and low‐functional lung regions. Furthermore, Liu et al.^24^ proposed a CNN‐based approach to synthesize Technegas SPECT ventilation images from non‐contrast 4DCT. They demonstrated, after post‐processing, Spearman's correlations of 0.73 and 0.71 for 10‐phase and 2‐phase 4DCT, respectively. Ten‐fold cross‐validation was used, achieving an average DSC across all folds of 0.83 for high‐functional lung regions, 0.61 for medium‐functional lung regions, and 0.73 for low‐functional lung regions. Subsequently, Grover et al.^25^ investigated the utility of CNNs for synthesizing Galligas PET ventilation images, demonstrating a mean Spearman's correlation of 0.58 and a mean DSC for high, medium, and low functional regions of 0.55. However, SPECT and PET have significantly longer acquisition times compared to CT imaging which facilitates acquisition within a single breath. This leads to the possibility of time‐delayed ventilation filling,^26^ reducing the relationship between structural and functional imaging modalities. Conversely, hyperpolarized gas MRI ventilation has an acquisition time spanning a single breath, similar to that of CT, leading to a potentially more accurate representation of ventilation at a specific point in time. Capaldi et al.^27^ has recently used a 2D UNet CNN to map free‐breathing proton MRI to ^3^He hyperpolarized gas MRI, achieving a Pearson correlation of 0.87 and a mean DSC of 0.90 and 0.37 for ventilated and defect lung regions, respectively. However, synthesizing hyperpolarized gas MRI directly from multi‐inflation CT has not yet been demonstrated.

Despite promising results achieved by DL synthesis techniques in multiple domains, there has been a lack of widespread adoption due to an inability to produce physiologically consistent results. Additionally, there is often a shortage of available data representative of a diverse population; to this end, several researchers have proposed the use of hybrid approaches that leverage computational modeling alongside data‐driven approaches, such as deep learning,^28^ precluding the requirement for large datasets. For example, hybrid physics‐ and model‐based approaches have been used in weather forecasting,^29^ earth surface modeling,^30^ and spatiotemporal dynamic systems evolution in robotics.^31^ Hybrid approaches have also been used for data generation in situations where there is limited data available.^32^

We hypothesized that a hybrid framework that integrates physiological‐based multi‐inflation level CT ventilation modeling and CNN‐based DL may generate accurate surrogate ventilation maps. Accordingly, we propose a hybrid model‐ and DL‐based framework, where conventional CT^HU^ models are used alongside structural inspiratory and expiratory CT scans as inputs to a CNN for functional lung image synthesis. In addition, we propose an automatic pipeline for predicting VLPs from the DL‐generated synthetic ventilation scans using CNN‐based segmentation. Due to the relatively small dataset, data‐driven approaches alone are unlikely to generate accurate synthetic ventilation images, especially in patients with significant ventilation defects. Therefore, the combination of data‐driven and physiological modeling approaches utilizes both methods’ benefits to produce physiologically consistent results, whilst also allowing features to be learnt from underlying patterns in the available data.

## 2 MATERIALS AND METHODS

### 2.1 Dataset

The dataset comprised paired inspiratory and expiratory CT and hyperpolarized ^3^He MRI scans for 47 patients originating from three clinical observational studies that were approved by the National Research Ethics Committee (REC). Lung cancer (*n* = 16) data was collected between 2015 and 2017 (REC: 14/LO/0481).^19^ Asthma (*n* = 12) data was collected between 2012 and 2013 (REC: 11/EM/0402).^33^ Cystic fibrosis (*n* = 19) data was collected between 2013 and 2014 (REC: 12/YH/0343).^34^

### 2.2 Image acquisition

Image acquisition details for CT and ^3^He MRI across the three studies are provided in Table 1. Additional image acquisition details are given in the subsequent sections.

**TABLE 1:** CT and hyperpolarized gas MRI acquisition details.

**Study**:	**Name**:	**Study 1**	**Study 2**	**Study 3**
	Disease:	Lung cancer	Asthma	Cystic fibrosis
	Total subjects:	16	12	19
**CT scans**:	Acquisition orientation:	Axial	Axial	Axial
	Dose mode:	Radiotherapy planning	High resolution	Ultra‐low dose (expiration) & low dose (inhalation)
	Breathing inflation:	FRC & FRC+1L	FRC & TLC	Inspiratory & expiratory breath‐hold
	Slice thickness:	2.5 mm	∼ 2.1 mm	2.5 mm
	In‐plane resolution:	∼ 0.98 × 0.98 mm^2^	∼ 0.8 × 0.8 mm^2^	∼ 0.6 × 0.6 mm^2^
	Tube voltage / Current:	120 kV / 315 mA	120 kV / 120 mA	80‐100 kV / 25−150 mA
**Hyperpolarized gas MRI scans**:	Hyperpolarized gas:	^3^He	^3^He	^3^He
	Dimension:	3D	2D	2D
	Sequence:	Balanced steady‐state free precession	Spoiled gradient echo	Spoiled gradient echo
	Acquisition orientation:	Coronal	Coronal	Coronal
	Breathing inflation:	FRC+1L	FRC+1L	FRC+1L
	Slice thickness:	5 mm	10 mm	10 mm
	In‐plane resolution:	∼ 4 × 4 mm^2^	∼ 3 × 3 mm^2^	∼ 3 × 3 mm^2^
	TR / TE:	1.9 / 0.6 msec	3.6 / 1.1 msec	3.6 / 1.1 msec
	Field of view:	40 cm	38.4 cm	30‐40 cm
	Flip angle:	10°	8°	8°
	Bandwidth:	±166.6 kHz	±63 kHz	±63 kHz
	Time‐difference:	Same day	< 4 days	Same day

Abbreviations: 1L, 1 liter; 2D, 2‐dimensional; 3D, 3‐dimensional; ^3^He, helium‐3; CT, computed tomography; FRC, functional residual capacity; MRI, magnetic resonance imaging.; SD, standard deviation; TE, echo time; TR, repetition time.

#### 2.2.1 CT acquisition

Study 1^19^: comprised 16 lung cancer participants. All participants underwent radiotherapy planning breath‐hold CT on a 16‐slice Lightspeed scanner (GE Healthcare, Princeton, New Jersey, USA); each acquisition was acquired within 15−20 s.

Study 2^33^: comprised 12 asthma participants. All participants underwent high‐resolution breath‐hold CT with a Sensation 16 CT scanner (Siemens, Forchheim, Germany).

Study 3^34^: comprised 19 cystic fibrosis participants. All cystic fibrosis participants underwent low dose inspiratory and ultra‐low dose expiratory non‐contrast CT imaging, following the protocol of Loeve et al.,^35^ on a GE Lightspeed VCT 64 CT scanner (GE Healthcare, Milwaukee, Wisconsin, USA). The CT scanner tube voltage was 80 kV for children weighing < 35 kg and 100 kV for those weighing 35 kg and above. Inspiratory scans were performed with a modulating tube current (max 150 mA) and expiratory scans were performed at a fixed current of 25 mA; as a result, expiratory scans were lower dose.

#### 2.2.2 MRI acquisition

All subjects underwent 3D volumetric ^3^He hyperpolarized gas MRI in the coronal plane at FRC+1L with full lung coverage at 1.5T on a HDx scanner (GE Healthcare, Milwaukee, Wisconsin, USA). Helium was polarized on‐site to around 25% polarization (GE Healthcare, Amersham, UK). Flexible quadrature radiofrequency coils were employed for transmission and reception of MR signals at the Larmor frequency of ^3^He (Clinical MR Solutions, Brookfield, WI, USA). An anatomical proton (^1^H) MRI in the same breath as ^3^He MRI was acquired for each patient. Details of this acquisition for each study are provided below:

Study 1^19^: Same‐breath ^1^H MRI scans were acquired at the same resolution as ^3^He MRI using the scanner's inbuilt body coil with a 3D spoiled gradient‐echo sequence. Repetition time/echo time were equal to 1.9/0.6 ms with a flip angle of 5° and ± 83.3 kHz bandwidth.

Study 2^33^: Same‐breath ^1^H MRI scans were acquired at the same slice thickness as ^3^He MRI with an in‐plane resolution of 3 × 6 mm^2^ using the scanner's inbuilt body coil with a 2D steady‐state free‐precision sequence. Repetition time/echo time was equal to 2.4/0.7 ms with a flip angle of 50° and ± 167 kHz bandwidth.

Study 3^34^: Same‐breath ^1^H MRI scans were acquired at the same resolution as ^3^He MRI using an eight‐element chest receiver array with a 2D steady‐state free‐precession sequence. Repetition time/echo time was equal to 2.9/0.9 ms with a flip angle of 50° and ± 250 kHz bandwidth.

### 2.3 Image segmentation

The Chest Imaging Platform (CIP)^36^ (Harvard, Massachusetts, USA) was used to generate segmentations of the lung parenchyma on inspiratory and expiratory CT scans. These segmentations were subsequently reviewed and manually edited by multiple experienced observers, specifically, B.A.T and J.R.A. Segmentation of the lung parenchyma from ^1^H MRI scans was conducted using spatial fuzzy c‐means clustering.^37^ ^1^H MRI segmentations were subsequently manually edited by two experienced observers, namely, B.A.T and P.J.C.H; both observers have a PhD in respiratory imaging.

### 2.4 Image registration

Inspiratory and expiratory CT scans were aligned using deformable image registration and subsequently registered to the spatial domain of ^3^He MRI via a corresponding anatomical ^1^H MRI scan as previously described.^19^, ^38^ Registration pipelines consisted of rigid, affine and diffeomorphic stages. All registrations were conducted using the advanced normalization tools (ANTs) registration framework^39^ based on parameters provided previously.^40^ For each patient, two registrations were performed:

1. Inspiratory CT to expiratory CT
2. Expiratory CT to ^1^H MRI (same‐breath as ^3^He MRI)

**FIGURE 1:**
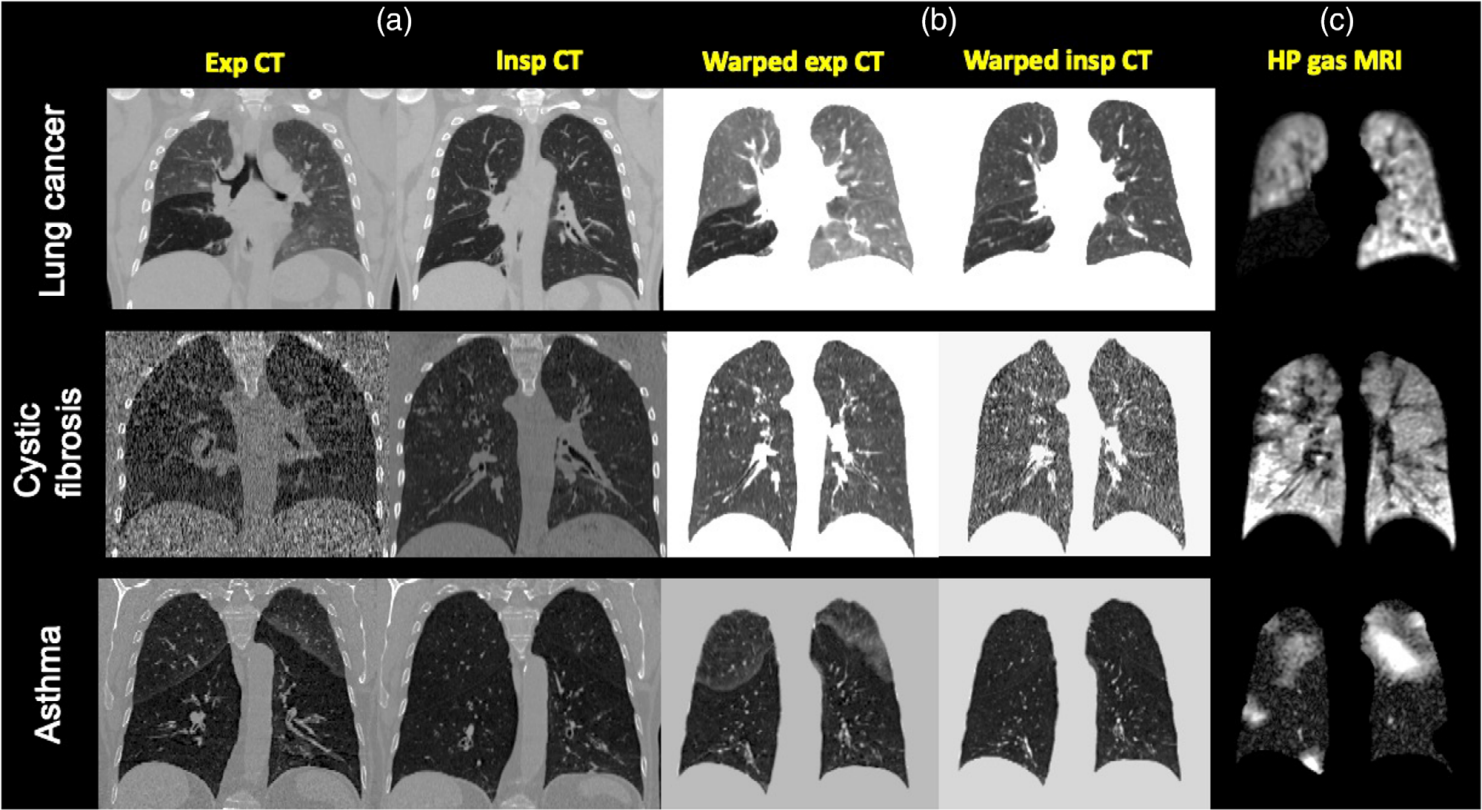
Example coronal slices for three patients with lung cancer, cystic fibrosis, or asthma of inspiratory and expiratory CT scans (a) before and (b) after deformable registration to the spatial domain of (c) hyperpolarized gas MRI.

Figure 1 shows example unregistered inspiratory and expiratory CT images with the corresponding warped CT images in the domain of ^3^He MRI. Registrations were quantitatively assessed for overlap using the DSC.^41^

### 2.5 CT ventilation modeling

CT‐based surrogate ventilation images were computed using the CT^HU^ model‐based metric originating from theory proposed by Simon et al.^42^ CTVI scans were generated at expiratory geometry and computed using voxel‐wise intensity differences in HU values based on the formulation by Guerrero et al.^9^ shown below:

(1) $$CT^{HU}=1000\frac{\bar{HU_{insp}}-HU_{exp}}{HU_{\exp\;}\left(1000+\;\bar{HU_{insp}}\right)}$$

where $\bar{HU_{insp}}$ represents the HU of voxels in the warped inspiratory scan that spatially correspond to voxels in the expiratory scan. $HU_{insp}\;$and $HU_{\exp\;}$represent the HU of inspiratory and expiratory voxels, respectively. CT^HU^ aims to measure the change in the fractional content of air, in a voxel‐wise manner, between expiratory and inspiratory phases.^43^ The method assumes that there is uniform air distribution in a given parenchymal voxel and that the observed change in lung density between respiratory phases is attributable solely to changes in ventilation. Tahir et al.^19^ previously demonstrated improved performance of the CT^HU^ metric over other CTVI metrics, such as CT^JAC^, via Spearman's correlation with hyperpolarized gas MRI on a subset of the data used in this study. Several CTVI works have employed various degrees of filtering to account for image noise and possible registration errors.^15^, ^19^, ^44^, ^45^ This has previously been used for post‐processing of CT^HU^ ventilation images in the range of 1 × 1 × 1 to 7 × 7 × 7 median filtering.^19^ To this end, we applied median filtering to CT^HU^ ventilation images across the whole lung region with a kernel size 6 × 6 × 1, due to the anisotropic resolution of ^3^He MRI.

## 3 DEEP LEARNING EXPERIMENTS AND EVALUATION

### 3.1 CNN architecture configurations

We evaluated four CNN configurations using either single‐channel or multi‐channel inputs as follows:

1. expiratory CT
2. inspiratory CT
3. expiratory CT + inspiratory CT
4. inspiratory CT + expiratory CT + CT^HU^ model

For each configuration, input feature maps constituting patches of 128 × 128 × 48 voxels were used due to memory constraints. Patches were fed into a 3D fully convolutional neural network with VNet architecture.^46^ The network consisted of convolutional steps containing between one and three convolutional layers with subsequent deconvolutional steps, enforcing the original input resolution. As demonstrated by Milletari et al.,^46^ each step is designed to learn residual functions by initially processing the first convolutional layer using a non‐linear activation function and subsequently replicating this output to the last convolutional layer within the step.^46^ Convolutional operations in the initial input block used two convolutional layers with 5 × 5 × 5 kernels and a stride of 1 followed by 2 × 2 × 2 kernels with a stride of 2 to reduce image dimensionality. For the multi‐channel configurations 3) and 4), we concatenated network blocks, combining the feature maps from spatially aligned inspiratory CT, expiratory CT and CT^HU^ modeling. This allowed the network to make use of concordant features represented across multiple inflation levels and modalities.^47^ The rest of the network consisted of four convolutional blocks that contained a varying number of convolutional layers with either 5 × 5 × 5 kernels with a stride of 1 or 2 × 2 × 2 kernels with a stride of 2, resulting in a maximum of 248 channels. Each convolutional operation employed a PReLU non‐linear activation function with valid padding. Subsequent deconvolutional blocks, with the same structure as the convolutional blocks, were used to reduce the number of channels. Fine‐grained feature forwarding introduced residual functions to corresponding convolution and deconvolution steps. The final output block made use of a 1 × 1 × 1 convolutional layer.

### 3.2 CNN training parameters

All hyperpolarized gas MRI, CT and CT^HU^ ventilation scans were masked by their respective lung parenchymal segmentations, thereby eliminating the effect of background voxels and allowing the network to focus on features within the lung parenchyma. All hyperpolarized gas MRI scans used in the dataset underwent pre‐processing to normalize image intensities to values between 0 and 1. Training data was augmented to reduce overfitting whilst still maintaining physiological plausibility. To do this, we employed constrained random rotations with limits −10° to 10° and scaling of −10% to 10%, where a different random rotation or scaling for each axis was applied at an interval within the defined limits above. The data augmentation method used does not increase the overall number of scans in the dataset; instead, each scan is given random scaling and rotation factors before being fed into the network. Therefore, the number of epochs can be increased as each time a scan is passed through the network, it is plausibly augmented by a different random factor at each epoch. Batch normalization was applied for each pass using a mini‐batch size of 2 with the aim of reducing covariate shift between network layers.^48^ The weights of the network were trained from scratch and initialized using Xavier initialization, representing a Gaussian distribution with mean of 0 and variance of 1/N, where N represents the number of weights and biases. A root mean square error (RMSE) loss was used to optimize the network employing Adam^49^ optimization with an initial learning rate of 1 × 10^−5^, reducing by a factor of 10 after 1500 epochs and trained for a total of 2150 epochs. L2 regularization with a decay of 0.00001 was used to penalize large network weights and minimize potential overfitting. Training and testing were performed using TensorFlow^50^ 1.15 and Python 3.6.^51^ Training was parallelized across four NVIDIA Tesla V100 GPUs each with 16GB of RAM.

Due to the somewhat limited size of the dataset, we employed 6‐fold stratified cross‐validation, generating six separately trained models tested on a random subset of 7 or 8 patients. The use of cross‐validation to increase the size of the testing set allowed for inferential statistical analyses to be conducted. Each model was stopped at 2150 epochs to constrain model training, mitigating overfitting. All DL configuration outputs were subsequently median filtered with a kernel size of 6 × 6 × 1 in line with the filtering applied to CT^HU^ ventilation images.

### 3.3 Quantitative evaluation

Synthetic ventilation images generated via the CT^HU^ method and DL configurations were quantitatively evaluated using both voxel‐wise and clinical metrics. Following previous works in the CTVI field and the VAMPIRE grand challenge, Spearman's correlation (𝜌) was selected as the primary evaluation metric.^22^ DL‐based methods were additionally assessed using the MSE metric. Further, based on voxel‐wise evaluation metrics, the clinical metric of VLP was computed on the best performing approach. Furthermore, regional localization of ventilated and defect lung regions was assessed via the DSC.

#### 3.3.1 Spatial correlation

The spatial correlations of the DL‐generated synthetic ventilation images and the CT^HU^ model against corresponding ^3^He MRI scans were assessed at full resolution using Spearman's 𝜌 on all voxels within the lung region, defined by the same‐breath ^1^H MRI lung segmentation. Spearman's 𝜌 quantifies the degree of monotonicity between any two ventilation images. It takes a range between −1 and 1 where 1 represents a perfect positive correlation and −1 represents a perfect negative correlation. Consequently, a Spearman's 𝜌 of 0 represents no correlation. Spearman's 𝜌 was used as the primary evaluation metric in this work.

#### 3.3.2 Mean square error

Quantitative performance was further evaluated for all DL‐based approaches using the voxel‐wise MSE metric. The MSE represents the mean square difference between estimated values and actual values across all voxels within the lung region. MSE is derived from the square of errors and, therefore, always takes a positive value with the MSE approaching 0 as the error concordantly decreases.

#### 3.3.3 Clinical evaluation

The quantitative biomarker of the VLP has been used extensively in the hyperpolarized gas MRI literature as a robust measure of lung function. VLP is calculated by comparing structural and ventilated lung segmentations to generate a percentage value of ventilated lung volume as follows:

(2) $$VLP\;\;\left(\%\right)=\left(\frac{ventilated\;lung\;volume}{total\;lung\;volume}\;\right)\;\times \;100$$

**FIGURE 2:**
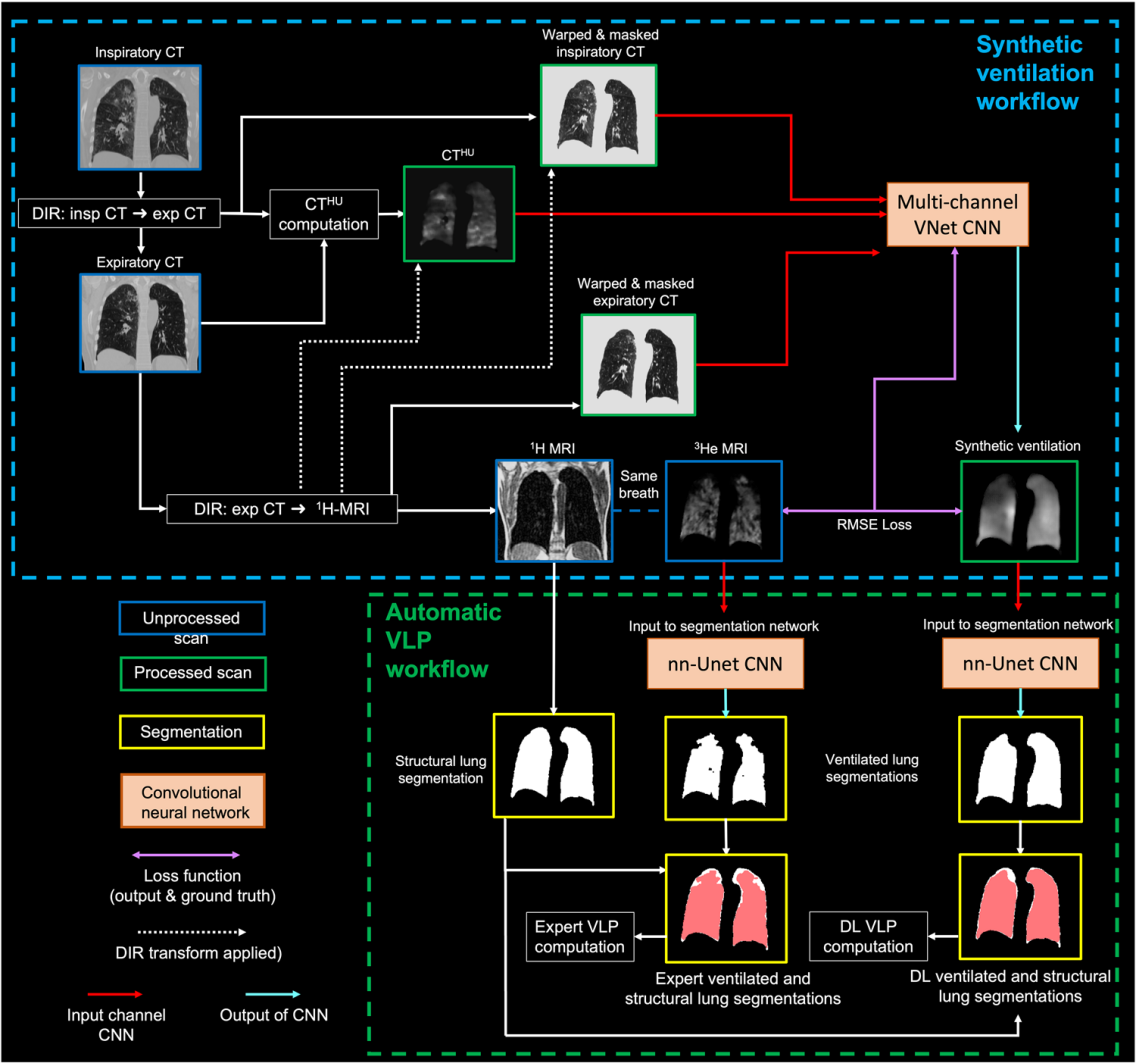
Hybrid model‐ and DL‐based synthetic ventilation workflow and accompanying automatic calculation of VLP.

In our clinical lung image analysis workflow, VLP values are derived from expert segmentations of hyperpolarized gas MRI for ventilated lung volumes and ^1^H MRI for total lung volume.^8^ In this study, we compared these expert VLP values to VLP values derived using the same ^1^H MRI expert segmentations for total lung volume and DL‐generated ventilated lung segmentations. We used a previously validated nn‐UNet CNN developed for automatic hyperpolarized gas MRI segmentation^52^ to segment synthetic ventilated lung regions from the best performing DL configuration and the CT^HU^ ventilation model. These segmentations were used to calculate VLP automatically without manual editing. Figure 2 depicts a high‐level description of the hybrid model/DL workflow and the automatic calculation of VLP values using DL and expert approaches. In addition to VLP values, DSC overlap values were computed between DL‐generated or CT^HU^‐generated ventilated lung segmentations and expert ^1^H MRI segmentations to define both ventilated and defect regions.

#### 3.3.4 Statistical analysis

Statistical analysis was performed using GraphPad Prism 9 (GraphPad, San Diego, CA). In this work, a p‐value < 0.05 was considered statistically significant. A one‐way repeated measures analysis of variance (ANOVA) test for multiple comparisons was used to determine differences between DL configurations for both voxel‐wise Spearman's 𝜌 and MSE. Post‐hoc paired *t*‐tests were used to assess differences in Spearman's 𝜌 between the CT^HU^ ventilation model and the four DL configurations compared to the reference ^3^He MRI ventilation scans. Kruskal‐Wallis tests were used to assess differences in Spearman's 𝜌 between the three studies contained within the dataset. Bland‐Altman analyses of bias were used to compare expert VLP values to DL‐derived and CT^HU^‐derived VLP values for the best performing DL‐based configuration. Paired *t*‐tests were used to assess differences in overlap of ventilated and defect lung regions for the best performing DL configuration and the CT^HU^ ventilation model.

## 4 RESULTS

### 4.1 Image registration

Registrations between inspiratory CT and expiratory CT, and expiratory CT and ^1^H MRI were evaluated using the DSC metric. All studies generated a median (range) DSC value exceeding 0.98 for inspiratory and expiratory CT, and 0.91 for expiratory CT and ^1^H MRI (see Table 2).

**TABLE 2:** Evaluation of overlap between inspiratory and expiratory CT and expiratory CT and ^1^H MRI.

Study	Insp CT to Exp CT Median DSC (range)	Exp CT to ^1^H MRI Median DSC (range)
Study 1	0.984 (0.969, 0.989)	0.963 (0.942, 0.973)
Study 2	0.986 (0.977, 0.988)	0.948 (0.930, 0.960)
Study 3	0.983 (0.970, 0.990)	0.919 (0.864, 0.955)

Median (range) DSC values are given for the three studies comprising the data used in this work.

### 4.2 Qualitative and quantitative evaluation

**FIGURE 3:**
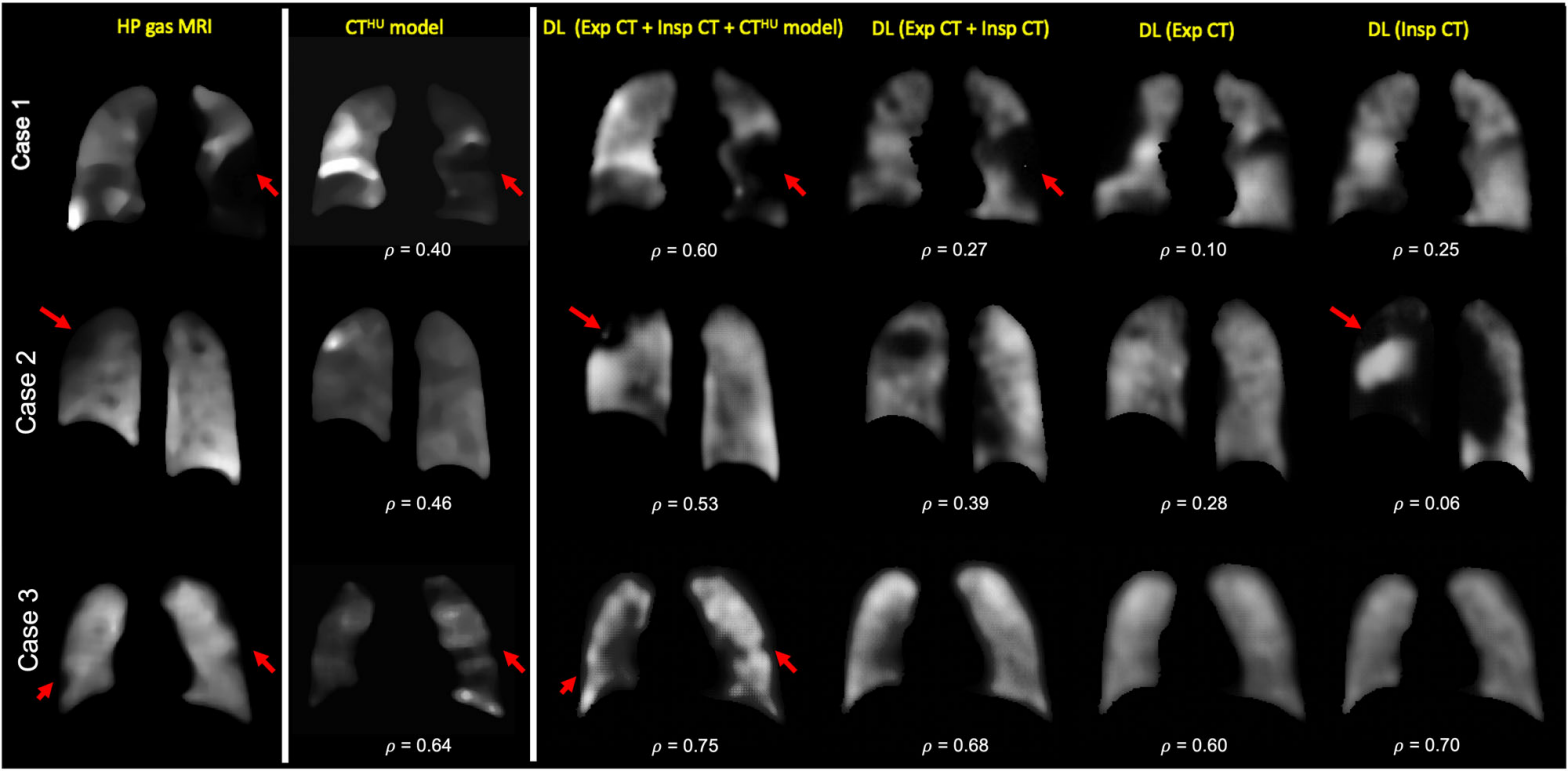
Example coronal slices from the CT^HU^ model and the four DL frameworks for three cases compared to ^3^He MRI. Spearman's 𝜌 values between each method and ^3^He MRI are provided. Red arrows demonstrate examples of replicated defects. Cases 1 and 2 are from lung cancer patients and Case 3 is from a cystic fibrosis patient.

Qualitatively, there are numerous examples of the hybrid DL‐generated synthetic ventilation images accurately replicating gross ventilation defects in the ground‐truth hyperpolarized gas MRI scans. Figure 3 shows qualitative spatial agreement between ^3^He MRI and synthetic ventilation approaches for three example cases. For the three cases displayed, the hybrid DL method, with inspiratory CT, expiratory CT and the CT^HU^ model as inputs, generated the highest Spearman's 𝜌 compared to the CT^HU^ model and all other DL configurations. For Case 1, the differences in performance between DL configurations demonstrate that when a singular structural image is used as an input, the resulting synthesized ventilation scan is unable to capture gross ventilation defects in the left lung; however, when the hybrid DL configuration is utilized, the resulting synthetic scan accurately captures gross ventilation defects which mirror defects observed in the hyperpolarized gas MRI scan.

Significant differences between methods were determined by a one‐way ANOVA test (*P* < 0.05). The hybrid method yielded statistically significant improvements in Spearman's 𝜌 compared to the CT^HU^ model with mean ± SD 𝜌 of 0.57 ± 0.17 versus 0.51 ± 0.22 (*P* = 0.003). Furthermore, this approach significantly outperformed all other DL configurations, which did not employ the CT^HU^ model as an input (*P* < 0.05). DL‐based approaches were additionally assessed using voxel‐wise MSE; the hybrid configuration generated the lowest MSE based on descriptive statistics. No significant differences were observed between the three best performing DL‐methods using the MSE metric. Table 3 summarizes the descriptive statistics for all methods across 47 patients via six‐fold cross‐validation.

**TABLE 3:** Descriptive statistics for the CT^HU^ model and DL configurations after combining the testing set performance via six‐fold cross‐validation.

	Spearman's 𝜌	MSE
Synthetic ventilation generation method	Mean ± SD	Mean ± SD
CT^HU^ model	0.51 ± 0.22	N/A
DL (expiration CT)	0.52 ± 0.20	0.024 ± 0.01
DL (inspiration CT)	0.47 ± 0.21	0.020 ± 0.01
DL (expiration CT + inspiration CT)	0.52 ± 0.19	0.020 ± 0.01
DL (expiration CT + inspiration CT + CT^HU^ model)	**0.57 ± 0.17**	**0.017 ± 0.01**

Mean ± SD Spearman's 𝜌 for the DL configurations and the CT^HU^ model are shown. Additionally, mean ± SD MSE are given for the DL configurations. The best 𝜌 and MSE values are shown in bold.

**FIGURE 4:**
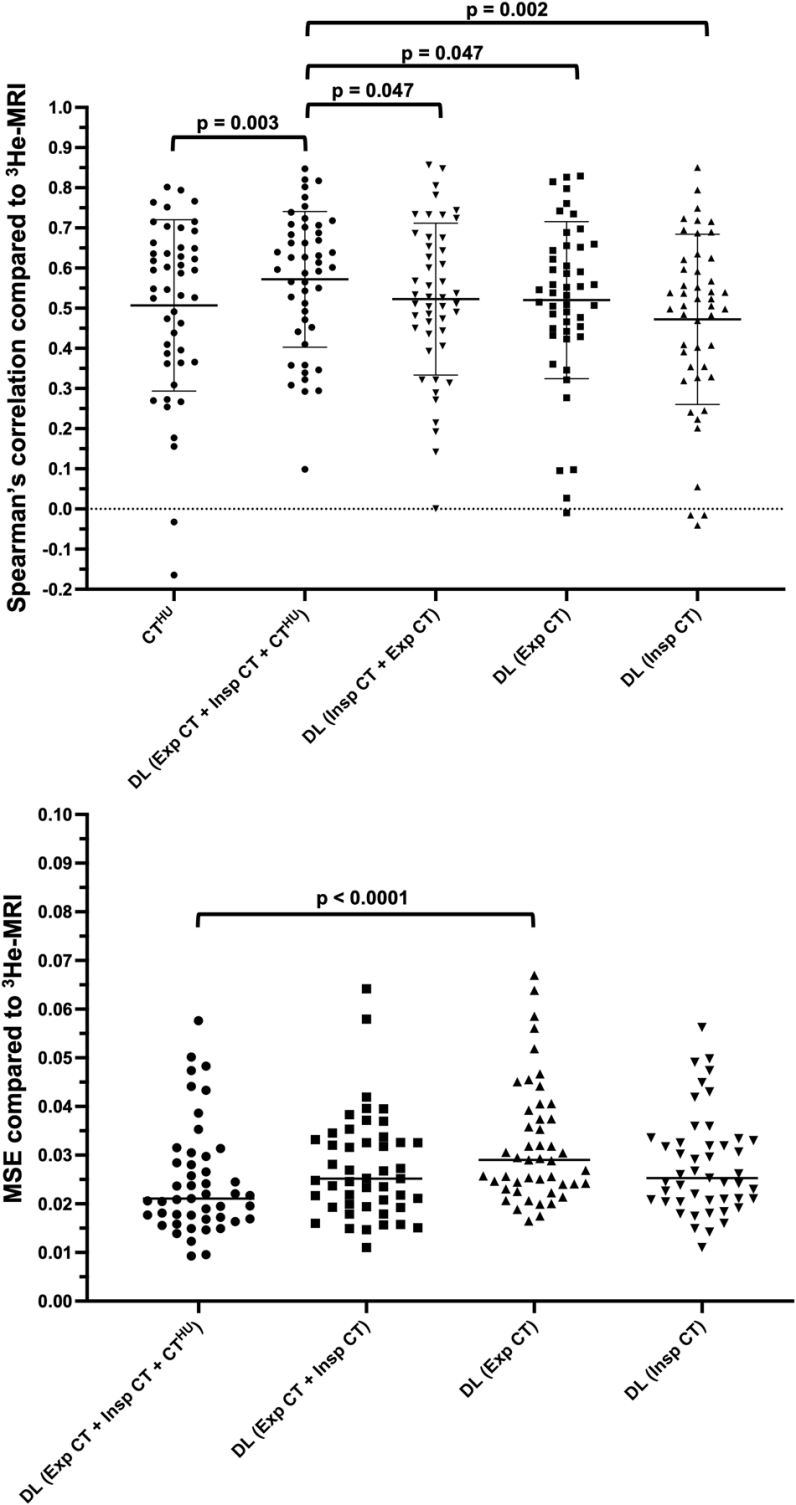
(Top) Spearman's 𝜌 values for synthetic ventilation scans derived from the CT^HU^ model and DL configurations. A paired *t*‐test compared CT^HU^ with the hybrid DL configuration. One‐way ANOVA tests compared Spearman's 𝜌 values for DL configurations. (Bottom) MSE values for synthetic ventilation scans derived from DL configurations. One‐way ANOVA tests compared MSE values for DL configurations. Only significant p‐values are provided.

Figure 4 shows Spearman's correlations between ^3^He hyperpolarized gas MRI for both the CT^HU^ ventilation model and DL‐based configurations; the proposed hybrid framework demonstrated significantly greater Spearman's correlations when compared to all other DL configurations and the CT^HU^ ventilation model. Additionally, MSEs between ^3^He hyperpolarized gas MRI and DL configurations are displayed, indicating minimal significant differences between DL configurations.

The dataset contains scans from three independent research studies with varying acquisition protocols from participants with varying pulmonary pathologies. No significant difference in Spearman's ρ between datasets was observed using the CT^HU^ ventilation model. A significant difference was observed between the Spearman's 𝜌 of Study 1 and Study 3 using the hybrid DL configuration (*P* = 0.03); no other significant differences were observed (Study 1 vs. Study 2, *P* = 0.93; Study 2 vs. Study 3, *P* = 0.51).

### 4.3 Clinical evaluation

**FIGURE 5:**
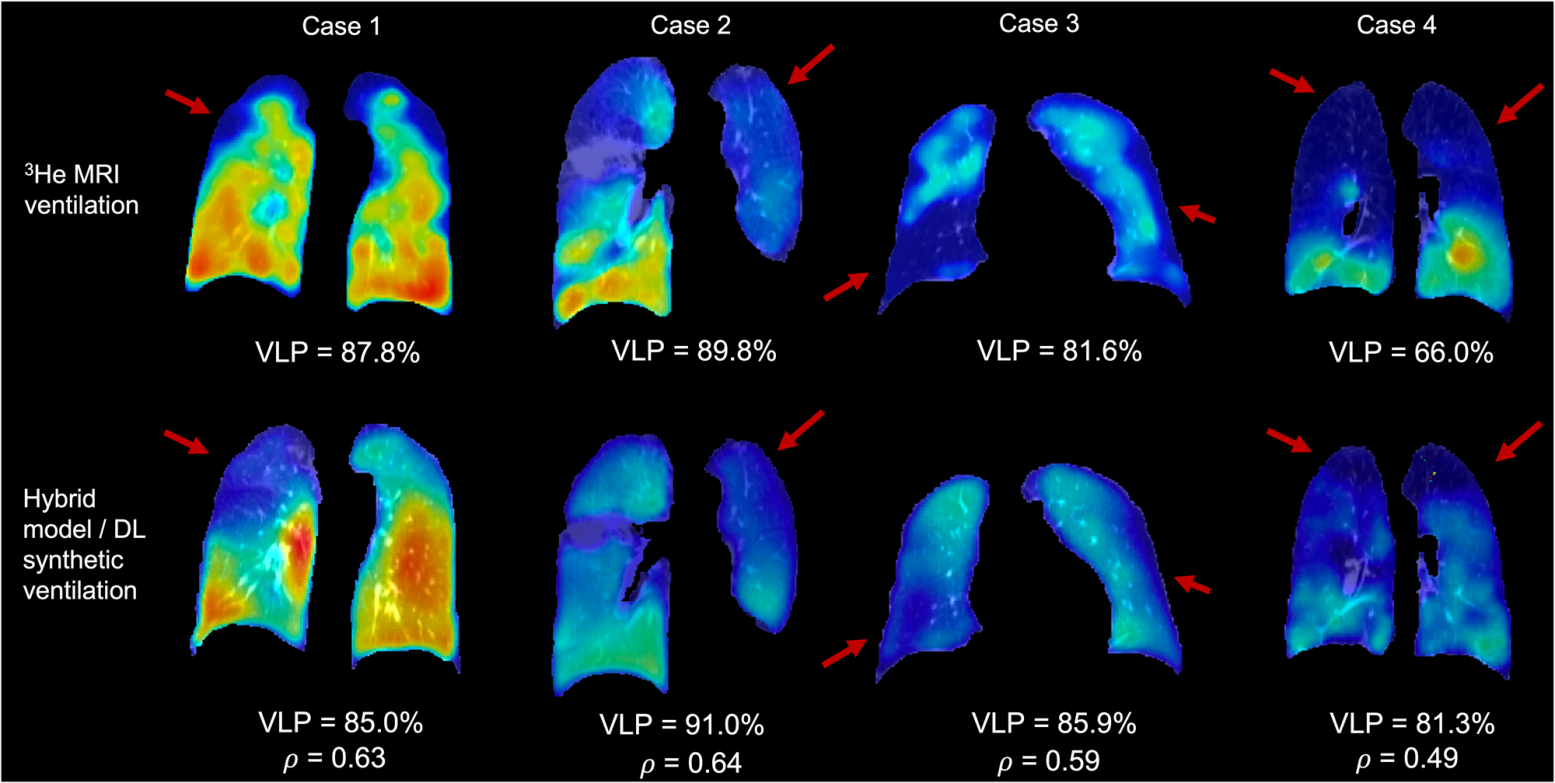
Fused ventilation (jet colormap showing minimum to maximum ventilation) and structural scans (grayscale) from four patients derived from either (top) ^3^He MRI and warped expiratory CT or (bottom) the proposed hybrid configuration and warped expiratory CT. All cases are from lung cancer patients with significant ventilation defects; red arrows indicate defects replicated in synthetic ventilation scans. VLP and Spearman's 𝜌 values are given.

**FIGURE 6:**
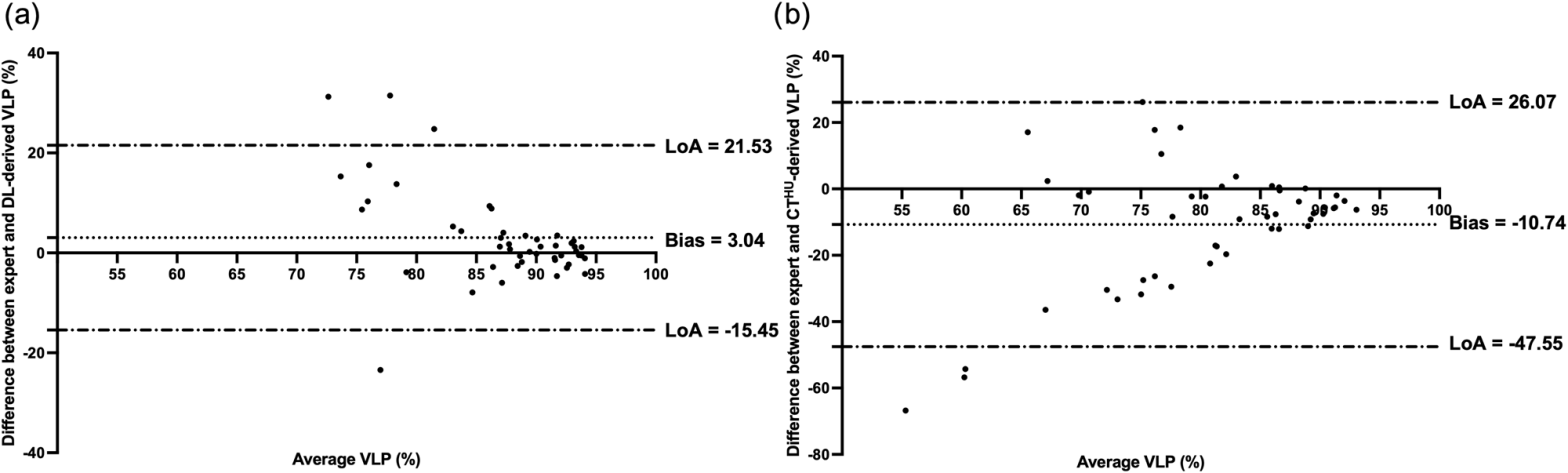
Comparison of VLPs derived from hyperpolarized gas MRI versus (a) the hybrid model/DL configuration and (b) the CT^HU^ ventilation model using Bland‐Altman analysis.

The hybrid model/DL configuration exhibited significant improvements in Spearman's 𝜌 when compared to all other methods investigated. Therefore, we further investigated this configuration using a clinical metric, namely, VLP. Using the workflow defined in Figure 2, we compared expert VLP values to those computed from synthetic ventilation scans generated by the hybrid configuration. Figure 5 shows fused structural and functional images with corresponding VLP values for four cases in the dataset. Cases with significant ventilation defects were chosen to illustrate the hybrid framework's ability to replicate gross defects. For example, Case 2 shows almost no ventilation signal in the left lung of the hyperpolarized gas MRI scan which is largely replicated in the output of the hybrid configuration. We used Bland‐Altman analyses of bias to compare VLP values derived from hyperpolarized gas MRI versus VLP values derived using the hybrid DL configuration and the CT^HU^ ventilation model as shown in Figure 6. The hybrid DL synthetic ventilation surrogates resulted in a bias of only 3.04% with limits of agreement (LoA) of −15.45% to 21.53% compared to the CT^HU^ ventilation model which produced a bias of −10.74% with LoA of −47.55% to 26.07%.

DSC values of ventilated and defect lung regions for the hybrid DL and CT^HU^ model are compared to expert ventilated and defect lung regions computed using hyperpolarized gas MRI (see Table 4). The hybrid DL configuration produced significantly greater DSC values for both the ventilated and defect lung regions, achieving a median (range) DSC of 0.946 (0.715, 0.977) and 0.483 (0.288, 0.743) for ventilated and defect lung regions, respectively.

**TABLE 4:** Median (range) DSC of the hybrid DL configuration and CT^HU^ ventilation model for ventilated and defect lung regions.

Region	Hybrid DL	CT^HU^
Ventilated lung	**0.946 (0.715, 0.977)**	0.903 (0.046, 0.956)
Defect lung	**0.483 (0.288, 0.743)**	0.426 (0.049, 0.730)

The best DSC values are shown in bold.

## 5 DISCUSSION

In this work, we proposed a hybrid model‐ and DL‐based framework, integrating CT^HU^ models of lung ventilation and structural, multi‐inflation CT as inputs to a VNet CNN capable of producing synthetic ventilation scans that correlated well with corresponding ground‐truth ^3^He MRI ventilation scans. To the best of our knowledge, this work represents the first use of DL to predict hyperpolarized gas MRI ventilation directly from multi‐inflation CT. As shown in Figures 3 and 5, the synthetic ventilation scans generated using the hybrid framework mimic moderate‐to‐large defects present in the corresponding ^3^He MRI scans. This has the potential to produce DL‐based synthetic ventilation scans from routinely acquired CT scans without exogenous contrast. Compared with conventional CT^HU^ modeling, the hybrid framework yields a statistically significant improvement in spatial correlation. The comparison with CT^HU^ ventilation surrogates is somewhat limited due to the inclusion of pulmonary vessels in CT^HU^ images. Commonly, vessels are excluded from CT^HU^ images; however, this adds a significant time‐consuming manual intervention step. The hybrid configuration developed here can potentially learn to accommodate pulmonary vessels without manual intervention through learning mechanisms. ^1^H MRI scans used in this study were acquired using spoiled‐gradient echo sequences; pulmonary vessels are significantly more challenging to identify using these sequences compared to balanced steady‐state free‐precession MRI^53^ or CT; hence delineating corresponding vessels in imaging modalities is a significant challenge. In addition to outperforming conventional CT^HU^ modeling, the hybrid configuration significantly outperformed all other DL configurations using Spearman's correlation, indicating the significant benefit of leveraging classical modeling and data‐driven approaches. The hybrid configuration's performance is further enhanced by harnessing a combination of structural and functional modalities. Functional CT^HU^ ventilation images have demonstrated moderate correlation with hyperpolarized gas MRI previously^19^; however, differences remain. By combining structural CT images at multiple inflations with CT^HU^ images, additional information contained within the structural images can be utilized to modify the predicted ventilation image via a deep learning approach. The measured Spearman's correlations of the CT^HU^ model and the hybrid configuration demonstrate some correlation with each other, but, crucially the inclusion of structural CT images in combination with the CT^HU^ images as inputs generated a significant improvement in Spearman's 𝜌 compared to the conventional CT^HU^ method or the DL configurations not integrating CTVI modeling. Although Spearman's 𝜌 was utilized as the primary evaluation metric, performance was also evaluated using the MSE. The MSE was not calculated for the CT^HU^ ventilation model as this model is directly derived from HU values, which have physiological meaning, limiting a direct quantitative comparison with hyperpolarized gas MRI where specific voxel intensity values are arbitrary and not consistent between scans. The MSE was calculated for all DL configurations, indicating minimal significant differences between DL configurations; this is potentially due to the MSE assessing specific values of intensity, compared to correlations between corresponding voxel intensities, and may be less important than correlated regions of low intensity.

We evaluated the hybrid framework on a diverse and challenging dataset using six‐fold cross‐validation. The dataset contained scans of patients with one of three lung pathologies, namely, lung cancer, moderate‐to‐severe asthma or mild cystic fibrosis. The scans were pooled from three separate clinical studies, resulting in a wide range of acquisition protocols in the dataset: high‐dose and low‐dose CT; different CT scanner types, settings and breathing maneuvers; 2D versus 3D ^3^He MRI; differences in in‐plane resolutions and slice thicknesses. The proposed hybrid framework exhibited some differences between studies present in the dataset, that is, between Study 1 and Study 3; however, it cannot be determined whether this variation in performance is due to differences in participant disease or the image acquisition parameters used. The lack of differences when comparing performance of the remaining study combinations indicates a level of robustness and generalizability to both disease and acquisition parameters. 6‐fold cross‐validation was employed, resulting in six separately trained models. This expanded the number of scans available for evaluation; however, the dataset remains relatively limited in size, containing only 47 patients. Future work will aim to expand the dataset further and investigate novel data augmentation techniques, including synthetic data generation. There is a potential that, as the amount of available representative scans increases, configurations excluding CTVI modeling may generate synthetic ventilation images that are more correlated with hyperpolarized gas MRI scans. In future work, if the dataset is expanded, we can assess whether the inclusion of the CTVI modeling still provides significant performance benefits.

The VNet CNN architecture was used due to its fully convolutional nature. Fully convolutional networks contain no fully connected layers and hence contain significantly fewer parameters than conventional networks with fully connected layers; this minimizes the network's ability to simply memorize scans within the training set, referred to as overfitting. The fully convolutional VNet not only reduces the overall number of parameters but also makes the number of parameters independent of image matrix size. Therefore, the network was trained and tested on scans with different matrices and acquisition protocols using fixed‐size patches of 128 × 128 × 48 voxels. We further reduced the possibility of overfitting using L2 weight regularization with a decay of 0.00001 to penalize large network weights.

Segmentations of ventilated lung volumes derived from hyperpolarized gas MRI and thoracic cavity volumes derived from structural ^1^H MRI segmentations have been extensively used in the literature to generate VLPs, an established biomarker of regional lung function.^7^ We demonstrated that VLPs derived from the proposed hybrid framework are comparable with ground truth VLPs from ^3^He MRI, producing a significantly reduced bias compared to the CT^HU^ method. Bland‐Altman analysis of bias, however, indicated that there was reduced accuracy in patients with more significant ventilation defects, resulting in higher predicted VLP values than the corresponding expert values. In addition to VLP analysis, synthetic ventilation scans were segmented using a DL‐based segmentation algorithm^52^ to provide regional localized comparisons of ventilated and defect lung regions. The hybrid DL configuration generated a median DSC of 0.95 for ventilated regions and 0.48 for defect regions, significantly outperforming the DSC achieved by the CT^HU^ method. Both VLP values and regional overlap values require the segmentation of synthetic ventilation scans and are, therefore, susceptible to biases in the segmentation algorithm used; the automatic segmentation method used here was trained to segment hyperpolarized gas MRI and not synthetic ventilation scans.^52^ There is limited consensus on the appropriate segmentation schema required for the delineation of ventilated and defect regions, resulting in an inability to produce accurate comparisons between research studies. It is possible that ventilated lung regions were overestimated during segmentation due to the less pronounced changes in ventilation heterogeneity. Further investigation to improve automatic segmentation of synthetic ventilation scans generated by the hybrid configuration could reduce these biases.

As previously demonstrated by Levin et al.,^54^ the minimum resolution of functional lung images need not be higher than the smallest pulmonary gas exchange unit, namely, the acinus, which has been estimated to be on the order of 10 × 10 × 10 mm^3^ in adult humans. They further indicate that resolutions of 20 × 20 × 20 mm^3^ may be appropriate due to the spatial clustering of most ventilation defects.^54^

Our study only investigates one CTVI modeling method, namely, CT^HU^; however, several other CTVI methods have been used in the literature. Subsequent research will aim to assess the differences in performance of the hybrid approach using classical CTVI metrics, such as CT^JAC^, and emerging metrics with more robust formulations.^10^, ^55^ One key consideration is the requirement of accurate registration between multi‐inflation CT and hyperpolarized gas MRI. Building a network capable of synthesizing ventilations scans independent of image registration would reduce the computational costs and time taken to generate synthetic images. Both the CT^HU^ metric and the proposed hybrid model rely on accurate registrations and, consequently, are susceptible to errors in cases where the registration is suboptimal. Removing this requirement would eliminate biases due to errors in registration.

A previous approach by Westcott et al.^56^ utilized texture analysis, feature selection and classical machine learning methods to generate synthetic lung ventilation maps from thoracic CT in COPD patients. They evaluated the synthetic ventilation maps using whole‐lung metrics; however, more accurate voxel‐wise evaluation metrics were not reported.

The ability to generate synthetic ventilation scans from CT has implications for several clinical applications, including functional lung avoidance radiotherapy^3^, ^4^ and treatment response mapping.^5^ Kida et al.^57^ has previously demonstrated that a Spearman's 𝜌 of ∼0.4 between CT^HU^ and SPECT images produces clinically indistinguishable radiotherapy plans. In this study, we observed correlations of ∼0.6 between the hybrid DL configuration and hyperpolarized gas MRI, indicating the former's potential clinical utility in functional lung avoidance radiotherapy. Synthesizing hyperpolarized gas MRI in comparison to other functional lung imaging modalities such as SPECT has several advantages, including enhanced spatial and temporal resolution and the lack of aerosol deposition artifacts or time‐delayed ventilation filling effects. CT is an integral part of almost every clinical lung imaging workflow and hence is readily available for most patients; therefore, synthetic ventilation from non‐contrast CT can provide patients with wider access to ventilation imaging worldwide.

## 6 CONCLUSION

We propose a hybrid model/DL framework to synthesize ventilation scans from routinely acquired non‐contrast multi‐inflation CT and classical CTVI modeling. We show that a synergy between model‐based CTVI and CNN‐based learning yields statistically significant improvements in performance compared with conventional CTVI modeling alone and other DL configurations that do not integrate modeling.

## CONFLICT OF INTEREST

The authors have no conflicts of interest to declare.

## Data Availability

The imaging datasets generated and/or analysed during the current study are not publicly available as they were generated as part of an industrial collaborative study that is still underway. Requests for data should be addressed to B.A.T.
